# Children’s Relationship With Their Pet Dogs and *OXTR* Genotype Predict Child–Pet Interaction in an Experimental Setting

**DOI:** 10.3389/fpsyg.2018.01472

**Published:** 2018-09-05

**Authors:** Darlene A. Kertes, Nathan Hall, Samarth S. Bhatt

**Affiliations:** ^1^Department of Psychology, University of Florida, Gainesville, FL, United States; ^2^Genetics Institute, University of Florida, Gainesville, FL, United States; ^3^Department of Animal and Food Sciences, Texas Tech University, Lubbock, TX, United States

**Keywords:** human–animal interaction, relationships, child, petting, dogs, *OXTR*, oxytocin receptor gene, oxytocin

## Abstract

Human–animal interaction (HAI) research has increasingly documented the important role of pet dogs in children’s lives. The quality of interaction between children and their pet dogs, however, is likely influenced by individual differences among children as well as their perceived relationship with their pet dog. Ninety-seven children aged 7–12 years and their pet dogs participated in a laboratory protocol during which the child solicited interaction with their dog, from which time petting and gazing were recorded. Children reported on their perceived relationship with the pet dog via interview. Children provided saliva samples, from which a polymorphism in the oxytocin receptor, *OXTR* rs53576, which has long been implicated in social behavior, was genotyped. The results showed that *OXTR* genotype and children’s perceived antagonism with the pet dog predicted the amount of petting, but not gazing, between children and their pet dogs. This research adds to the growing body of HAI research by documenting individual differences that may influence children’s interactions with animals, which is key to research related to pet ownership and understanding factors that may impact therapeutic interventions involving HAI.

## Introduction

Human–animal interaction (HAI) research has increasingly documented the important role of pet dogs in providing social support to children (e.g., Friedmann et al., 1983; Kotrschal and Ortbauer, 2003; Anderson and Olson, 2006). However, the bulk of this research has been descriptive or correlational in nature, with long-standing concerns about methodological rigor (Griffin et al., 2011) and few well-controlled laboratory experiments that afford greater confidence to the validity of results (Wilson and Barker, 2003). Our group has previously reported that children randomly assigned to experience a standard laboratory stressor accompanied by their pet dog for social support reported feeling less stressed compared to children who completed the stressor with their parent present or with no social support (Kertes et al., 2017). Among the children who underwent the stressful experience with their pet dog, those who naturally solicited their dog to be stroked or petted had lower levels of the stress-sensitive hormone cortisol compared to children who engaged their dog less.

Indeed, petting, and to a lesser extent gazing, have been suggested as potential mechanisms by which HAI impact humans by altering their emotional and physiological state (e.g., Odendaal and Meintjes, 2003; Shiloh et al., 2003). Among adults, petting is associated with reduced perceived stress or anxiety (Shiloh et al., 2003; Barker et al., 2005), increased immunoglobulin A (Charnetski et al., 2004), lower heart rate or blood pressure (Jenkins, 1986; Vormbrock and Grossberg, 1988; Demello, 1999; Handlin et al., 2011), and changes in β-endorphins, prolactin, β-phenylethylamine, oxytocin, cortisol, and dopamine (Odendaal, 2000; Barker et al., 2005). Adult owner-pet gazing has been linked with increased oxytocin levels (Nagasawa et al., 2009, 2015). Among children, petting during child–dog interaction has been associated with lowered cortisol stress response (Kertes et al., 2017) and positive affect (Kerns et al., 2018). The vast majority of research on HAI with children has focused on dog presence, which has been linked with reduced blood pressure (Friedmann et al., 1983) perceived stress (Kertes et al., 2017), enhanced emotional stability in the classroom (Anderson and Olson, 2006), increase social interaction, and decrease aggression and hyperactivity (Kotrschal and Ortbauer, 2003), and reduced distress to a routine medical procedure (Vagnoli et al., 2015).

Its potential benefits notwithstanding, the degree to which children’s interactions with their pet dogs spontaneously include petting and gazing may be influenced by individual differences in children’s perceived relationship with their pet dog. To date, the majority of research on pet owners’ feelings toward their pets have centered on positive emotions (Johnson et al., 1992; Cromer and Barlow, 2013). This area of research has shown that children and adults alike report strong positive feelings toward their pet dogs (Serpell, 1996; Daly and Morton, 2006; Kurdek, 2008). Noticeably absent from most HAI studies is the role of perceived negative aspects of the child–pet relationship, such as feeling annoyed with or hassled by interactions with the pet. A more complete evaluation of effects of children’s feelings toward their pet necessarily involves inclusion of both positive and negative components to children’s perceived relationships (e.g., DeRosier and Kupersmidt, 1991; Furman and Buhrmester, 1992; Shantz and Hartup, 1992; Van Horn and Cunegatto, 2000; Moilanen and Raffaelli, 2010).

Another factor that may contribute to individual differences in children’s interactions with their pet is variability within the oxytocinergic system. Oxytocin is a hormone and neuromodulator shown to be involved in a variety of social behaviors (Carter, 2014). Oxytocin is linked with affiliative behavior (Insel, 1992), formation of social bonds (Lim and Young, 2006), and responses to stressful social situations (Neumann et al., 2000; Kirsch et al., 2005).

Endogenous circulating oxytocin effects are influenced by actions at the oxytocin receptor. This receptor is encoded by the gene *OXTR*, which is variably expressed across individuals. Among the most commonly studied genetic polymorphisms at *OXTR* is rs53576, involving a guanine (G) to adenine (A) substitution. Genetic variation at this locus has been associated with both prosocial and negative behaviors. For example, A carriers, (i.e., individuals with the AA or AG genotype), compared to those with the GG genotype, have demonstrated lower levels of interpersonal empathy (Rodrigues et al., 2009; Smith et al., 2014), trust (Krueger et al., 2012), as well as lower self-esteem (Saphire-Bernstein et al., 2011), and higher negative affect and loneliness (Lucht et al., 2009). Among adolescents, A-carriers are reported to be less responsive to parental support (Smearman et al., 2016). Consistent with the differential susceptibility hypothesis (Belsky et al., 2009), it has been suggested that *OXTR* rs53576 may be one of a set of susceptibility loci in the genome, whereby genetic variation influences an individual’s sensitivity to the social environment (Kim et al., 2010).

Notably, the extensive literature examining *OXTR* rs53576 in relation to social behavior has focused exclusively on human social interaction. To date, there are no published studies examining *OXTR* genotype with respect to children’s interaction with animals. This is notable because interaction with and ownership of lay or trained therapy dogs is increasingly becoming a mainstay of clinical interventions for children with anxiety disorders, autism spectrum diagnoses, or a history of maltreatment, on the assumption that interacting with animals is particularly beneficial for individuals for whom social interactions are challenging (Nimer and Lundahl, 2007). The present report is the first to assess whether the *OXTR* genotype among children is related to HAI.

This study focused on typically developing children in middle childhood (aged 7–12 years). During this developmental period, the amount of time children spend with parents declines dramatically compared to earlier ages (Lam et al., 2012). Although parents continue to be important social partners, in middle childhood, children begin to rely on a broader network of social support figures compared to earlier ages, including pets (Bryant, 1985).

The purpose of the present study was to test whether children’s perceived relationships (including both positive emotional support and negative interactions) along with child genotype at the *OXTR* rs53576 polymorphism predict directly observable child–pet interaction. To achieve this aim, we assessed two essential elements of child–pet interaction, petting, and gazing, via direct behavioral observation in the context of a controlled laboratory environment with minimal distractions. Based on the extant literature linking *OXTR* rs53576 genotype to social behavior, we expected that children who are A-carriers would differ from those with the GG genotype with respect to the amount of time spent petting and gazing with their pet dogs. Because of the complex associations of the *OXTR* genotype with respect to social behavior, and the fact that this is the first study to examine the rs53576 polymorphism with respect to HAI rather than human social interactions, we did not specify *a priori* directional predictions for the *OXTR* genotype. With respect to children’s relationships, we anticipated that children’s perceived relationship with their pet dogs, as reflected in higher levels of perceived support and lower levels of perceived negative interactions, would be associated with higher levels of petting and gazing with their pet dogs. Children were also asked about their perceived relationship with the mother, which was included on conceptual grounds for their key role in social emotional development, and to evaluate whether child–parent relationships were related to child–pet interactions.

## Materials and Methods

### Participants

Participants were 97 children (49 boys; 48 girls) accompanied by a parent (81% mother) and pet dog. Participating families were recruited through locally distributed mailings, flyers, and radio and TV advertisements. Interested families contacted the research lab and were screened for eligibility. To be eligible for the study, children could not have any diagnosed physical or behavioral health conditions, and the pet dog must have lived with the family for at least 6 months and have no history of aggression. If multiple dogs resided in the home that met inclusion criteria, the family selected one dog based on the child–pet relationship to accompany the child to the research lab.

Child age ranged from 7 to 12 years (*M* = 10.3 years, *SD* = 1.32) Child ethnicity was reported by parents as follows: 11% Hispanic; 89% non-Hispanic. The majority of the sample was White (84%), with the remainder reporting their race as follows: 7% two or more races; 3% Latino; 2% Native American; 2% African American, and 2% Asian.

### Procedure

Procedures were approved by the University of Florida Institutional Review Board and the Institutional Animal Care and Use Committee. All procedures took place in three adjacent rooms – a waiting room, interview room, and experimental testing room – at the research laboratory at the University of Florida. Children were aware of their parent’s and dog’s location at all times. All rooms were temperature controlled and water was available for the dog.

At the start of the study visit, parents provided written informed consent in the waiting room. The study was also explained to children verbally for purposes of oral assent. A trained dog handler brought the dog to the experimental testing room to familiarize the dog to the room and study personnel. Then, the child accompanied an experimenter to the interview room, decorated in child-friendly décor while the dog remained with the parent in the waiting room.

In the waiting room, parents completed questionnaires providing basic demographic information on their child, family, and pet dog. Parents also provided information about the breed of the dog, which was subsequently classified into breed groups. Children’s pets included lap/toy dogs (*n* = 31), sporting breeds (*n* = 20), herders (*n* = 18), terriers/ratters (*n* = 12), bully/fighting breeds (*n* = 11), and unknown mixes (*n* = 5). A research assistant was present throughout to answer any parent questions.

#### Children’s Perceived Relationships

In the interview room, children completed an experimenter-assisted questionnaire about their relationships with their mother and their pet dog using the Network of Relationships Inventory (NRI) (Furman and Burmheister, 1985). The original NRI, comprised of 21 items, was designed and has been validated for assessing relationship qualities across a broad variety of social relationships, including but not limited to parents, teachers, and peers. An example item from this measure is, “How often do you tell this person everything that you are going through?” The measure contains 10 subscales typically collapsed into two broader scales, termed Support and Negative Interactions. We have previously evaluated the NRI among children owning pet dogs to determine the relevance of items for assessing child–pet relationships. With the exception of two subscales, Instrumental Aid and Conflict, the remaining subscales were retained as applicable to child–pet relationships (Hall et al., 2016).

The NRI items tapping relationship with the mother was scored as recommended by the scale developers (Furman and Burmheister, 1985). Then, the overarching dimension of Support was created by computing the mean of the items on the subscales Companionship, Intimate Disclosure, Nurturance, Affection, Admiration, Reliable Alliance, and Instrumental Aid. The dimension of Negative Interactions was computed as the mean of the scores on Conflict and Antagonism.

The NRI pet items were subjected to a principal component analysis (PCA) to determine whether a two-dimension solution was appropriate with the more limited set of subscales assessed for child–pet relationships. As described in the Section “Results,” a two-dimension solution was deemed appropriate for the data, and therefore the subscale means were computed and averaged into the two broad dimensions as follows: Support (Companionship, Intimate Disclosure, Nurturance, Affection, Admiration, Reliable Alliance) and Negative Interactions (Antagonism).

#### Behavioral Assessment of Child–Pet Interaction

The child and pet dog were brought to the experimental testing room for behavioral assessment of interaction between the child and pet. Specifically, this assessment measured the proportion of time the child and dog spent interacting while the child was sitting quietly in a room (4.5 m by 3 m) that contained a chair, desk, and lamp for 10 min. This task was based on components of past sociability tests (e.g., Barrera et al., 2010; Jakovcevic et al., 2012), but were simplified such that the child could implement the protocol with brief instruction. The child sat in a chair in the center of a 1 m radius semi-circle marked with tape on the floor. The dog handler brought the dog to the opposite end of the room, where the dog was able to greet a second observer for approximately 1 min before beginning the task. The child was asked to direct attention to the dog, and call the dog over twice while remaining seated, once at the beginning of the 10 min session and again after the first 5 min. The child was asked to otherwise remain neutral unless the dog entered the semi-circle. If the dog entered the circle the child was told to interact with it as if they were at home, to capture natural variability in child–pet interaction. During the assessment, the handler and observer stood along the wall opposite of where the child was seated for an unobstructed view of the child–pet interaction.

The handler and the observer, both trained in coding dog behaviors, scored each session live on two dimensions: gazing and petting. Each behavior was scored using partial-interval recording by breaking the 10 min session into 120 5 s. epochs. If a target behavior occurred during that epoch, the interval was scored. The percentage of epochs during which a target behavior occurred was averaged across the scorers. Gazing was defined as the percentage of intervals in which the dog and child were facing each other. Petting was defined as the percentage of intervals in which the child made contact with the dog with their hand. Inter-class correlations among the two coders was 0.85 for gazing and 0.99 for petting.

#### Genotype Assessment

Children were asked to provide a 4 mL saliva sample by passive drool into Oragene-DNA (OGR-500) saliva collection tube (DNA Genotek, Kanata, ON, Canada), which was stored at room temperature until the DNA extraction step. DNA extraction was performed using our lab’s standardized protocol. Briefly, 750 μl of the content from OGR-500 tube was incubated at 50°C in a GeneMate dry bath (Bioexpress, Kaysville, UT, United States) for 2 h, followed by incubation on ice for 10 min and centrifugation at 21,100 ×*g* for 10 min. The DNA was precipitated by transferring the supernatant to a tube containing 750 μl of ethanol, mixing the content gently, incubating at room temperature for 10 min, and centrifuging at 16,000 ×*g* for 3 min. The DNA pellet was washed using 70% ethanol, dried at room temperature, dissolved in 100 μl TE buffer and stored at -80°C. The DNA quality and quantity was measured using NanoDrop 2000 spectrophotometer (Thermo Fisher Scientific, Wilmington, DE, United States). Genotyping was performed using TaqMan Genotyping Master Mix (Applied Biosystems, Foster City, CA, United States), TaqMan SNP Genotyping assay (Applied Biosystems, Foster City, CA, United States) for *OXTR* (rs53576), with the StepOnePlus real time PCR system (Applied Biosystems, Frederick, MD, United States), according to the manufacturer’s instructions. PCR was performed using 10:l reaction mix with 1.5 ng DNA and the following cycling conditions: 60°C for 30 s, 95°C for 10 min, 40 cycles of 95°C for 15 s and 60°C for 1 min, 60°C for 30 s. Allelic discrimination was performed using StepOne v.2.1 (Applied Biosystems, Frederick, MD, United States).

### Statistical Analysis

Statistical analyses were conducted in R version 3.3.2 (R Core Team, 2016). A hierarchical regression framework with backward selection was utilized to test for significant predictors of two dimensions of child–pet interaction, with predictors of petting and gazing tested in separate models. Tested predictors included demographics, relationship qualities, and child genotype.

## Results

### Evaluating the NRI to Assess the Child–Dog Relationship

We first calculated the mean scores for each subscale for the child–dog relationship. We then conducted Principal Component Analysis on the scaled scores of each subscale. Two components explained 71% of the variance (55 and 16% respectively). The loadings of each component are shown in **Table 1**. Inspection of the loadings suggests a two-factor solution identical to *Support* and *Negative Interactions* used for computing summary scores for the child–mother relationship. This suggested that these two summary scores can be computed similarly for the child–dog relationship.

**Table 1:** Principal component analysis (PCA) loadings of dog NRI subscales.

	PC1	PC2
Companionship	**-0.45**	0.19
Antagonism	0.14	**0.98**
Intimate	**-0.41**	-0.05
Nurturance	**-0.45**	0.08
Affection	**-0.43**	0.01
Reassurance	**-0.47**	0.08

*Loading absolute values >0.4 are in bold.*

### Descriptives

Descriptive statistics of child-reported relationship qualities and behavioral observation of child–pet interaction are shown in **Table 2**. Genotype assessment of rs53576 yielded the following genotypes: 41% GG, 53% AG, and 6% AA. These proportions are comparable to other U.S.-based studies (see for review Luo and Han, 2014). As is common in analysis of rs53576, the AG, and AA genotypes were combined into one genotype group, termed A-carriers, yielding two genotype groups for analysis, GG homozygotes, (41%) and A-carriers (59%).

**Table 2:** Descriptive statistics for child self report relationship qualities and behavioral observation.

	Mean	*SD*
**Children’s reported relationships**		
Support from mom	3.78	0.66
Negative interactions with mom	2.02	0.81
Support from dog	3.91	0.68
Negative interactions with dog	1.52	0.81
**Behavioral observation of child pet interaction**		
Percent time spent petting	50.00	31.04
Percent time spent gazing	19.85	14.84

### Analysis of Petting

**FIGURE 1:**
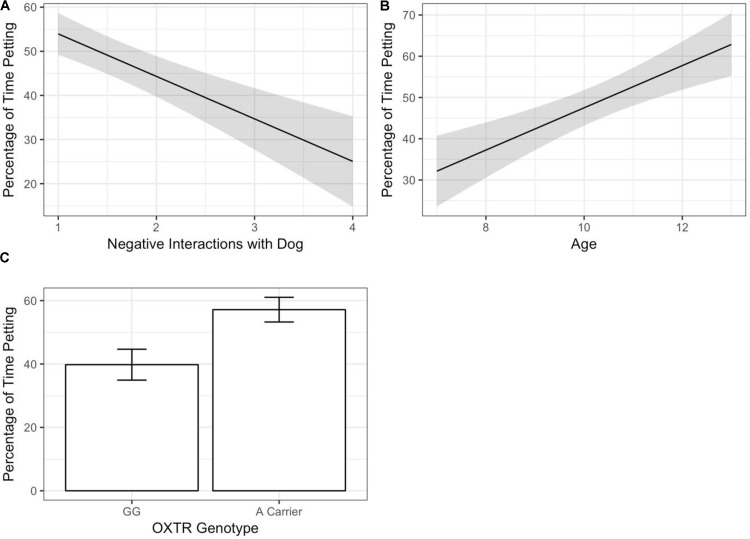
The proportion of time spent petting during a child–dog interaction task was independently predicted by child age, child genotype, and children’s self-reported relationship with the pet dog. **(A)** Higher levels of child-reported negative relationships with the pet dog was associated with less petting. The relationship effect is shown averaged across child genotype and illustrates the overall main effect for the child–pet relationship. The regression line shows the final model prediction with the shaded area indicated standard error of the mean. **(B)** Older child age is associated with more time spent petting. Lines shows reduced model prediction for each genotype and shading indicates standard error of the mean. **(C)** Children who are A-carriers at the *OXTR* rs53576 genetic polymorphism, compared to GG homozygotes, engaged in more petting during the child–pet interaction.

Predictors of child–dog petting during the child–pet interaction task were determined via regression analyses using backward selection to obtain a reduced model with the strongest predictors. An initial model included all predictors (demographic covariates, child self-reported relationship qualities, and child genotype). The initial model suggested that petting was associated with child age [*F*_(1,84)_ = 4.18, *p* = 0.044], *OXTR* rs53576 genotype [*F*_(1,84)_ = 6.15, *p* = 0.015], and Negative Interactions with the dog [*F*_(1,84)_ = 6.31, *p* = 0.014]. Specifically, more petting was observed with older child age, *OXTR* rs53576 A-carrier status, and lower child-reported Negative Interactions with the pet dog (see **Figure 1**). Petting was not associated with child sex [*F*_(1,84)_ = 0.37, *p* = 0.54], dog breed group [*F*_(5,84)_ = 1.47, *p* = 0.21], child-reported Support from dog [*F*_(1,84)_ = 1.09, *p* = 0.300], or either of the two child-reported measures of relationship quality with the mother [*F*_(1,84)_ = 0.53–1.21, *p* = 0.27–0.47]. To obtain the most parsimonious model for our dataset, the model was subjected to backward selection using the *Step* routine, which identifies the most parsimonious model based on Akaike’s information criterion (AIC; see **Table 3** for AIC values at each regression step). All three significant predictors – child age, child genotype, and the Negative Interactions dimension of the child-reported relationship measure – were retained in the final model, as shown in **Table 4**.

**Table 3:** Akaike’s information criterion (AIC) for each step in the regression predicting petting using backward regression.

Step	*df*	Deviance	Residual *df*	Residual deviance	AIC
Full model	NA	NA	84	69857.28	664.21
Dog support	1	9.91	85	69867.19	662.22
Child sex	1	242.34	86	70109.53	660.56
Breed	5	6680.03	91	76789.56	659.39
Negative interaction mom	1	373.48	92	77163.05	657.86
Mom support	1	517.13	93	77680.18	656.51

*Values are shown for each variable successively dropped from the model to achieve the best fitting results.*

**Table 4:** Final regression model of significant predictors of child–dog petting during behavioral observation.

	Estimate	Std. Error	*t*-value	*P*-value
Intercept	4.63	23.06	0.20	0.84
Age	5.12	2.30	2.23	0.03
OXTR (GG vs. A-carrier)	12.66	6.16	2.06	0.04
Negative interactions with dog	-9.63	3.81	-2.52	0.01

### Analysis of Gazing

A comparable regression model was created for gazing during the child–pet interaction. The initial model including all predictors revealed that none of the demographic, genotype, or relationship quality variables was significantly associated with gazing (*F*’s = 0.01–2.14, *p*’s > 0.15). The backward selection procedure was implemented to yield the most parsimonious model. The final model of gazing during the child–pet interaction task included only child age as a predictor, however, the association was not statistically significant (*F* = 2.28, *p* = 0.13; see **Table 5**).

**Table 5:** AIC for each step in the regression predicting gazing using backward regression.

Step	*df*	Deviance	Residual *df*	Residual deviance	AIC
Full model	NA	NA	88	20320.92	536.443
Support from mom	1	1.83	89	20322.76	534.44
Negative interactions mom	1	6.66	90	20329.42	532.48
OXTR Genotype	1	13.87	91	20343.28	530.54
Negative interactions dog	1	35.40	92	20378.69	528.71
Breed	1	36.44	93	20415.13	526.88
Child sex	1	47.90	94	20463.02	525.11
Support from dog	1	188.62	95	20651.65	524.00

*Values are shown for each variable successively dropped from the model.*

## Discussion

The present study was the first to test whether the *OXTR* genotype and children’s perceived relationships with their pet dogs are related to HAI, specifically, petting and gazing. The research design simulated a common, naturally occurring HAI, in which human owners call over their pets, within the context of a controlled laboratory experiment with minimal distractions. On average across children, the total time spent petting was approximately 50% of the 10 min interaction period. The results showed that variation at the *OXTR* polymorphism rs53576 was associated with the proportion of time spent petting during child–pet interactions. Specifically, A-carriers engaged in more petting than children with the GG genotype. This observation is noteworthy given that *OXTR* rs53576 has previously been suggested as a genetic locus associated with sensitivity to the social environment. Prior research with typically developing children has demonstrated that A-carrier youth are less responsive to parental support (Smearman et al., 2016) and to social consequences of peer relational aggression (Kushner et al., 2017), and show lower levels of interpersonal empathy (Rodrigues et al., 2009; Smith et al., 2014), trust (Krueger et al., 2012), and self-esteem (Saphire-Bernstein et al., 2011). This may be relevant to growing trend of incorporating HAI into behavioral therapy with children for whom human social interactions are challenging (Nimer and Lundahl, 2007; Silva et al., 2018). Although little empirical research has been conducted in this area, there is preliminary evidence that dogs may be preferred social partners for such children. Children with autism, a neurodevelopmental disorder in which social deficits are common, prefer to interact with a dog over another person or toy (Prothmann et al., 2009). Children with anxiety disorders tend to spend long durations interacting with a pet dog but tend to engage in fewer interactions with another person compared to children with other behavioral health problems (Prothmann et al., 2005). Although highly speculative, our results contribute to emerging evidence that pet dogs may be an important source of social interaction for children that have difficulty in other social environments.

The results of this study also demonstrated that children’s self-reported negative interactions in the context of their relationship with their pet was related to the proportion of time spent petting the dog. Specifically, higher levels of antagonism, reflecting children’s reports that they and their dog hassle each other, annoy each other, and “get on each others’ nerves,” spent less time engaged with petting. Children’s perceptions of support, reflecting items tapping aspects of affection, companionship, and other positive features, were not associated with the proportion of time spent petting. Of note, this was the first study that simultaneously assessed both positive and negative components of children’s relationships with their pet dog. Psychometric data from the principal components analysis demonstrated that children’s responses about positive and negative relationship qualities were distinct measurable aspects of the child–dog relationship that paralleled the relationship dimensions measured for the child–parent relationship. The observation that negative interactions, and not support, was associated with petting speaks to the need to incorporate both positive and negative aspects of relationships in HAI research.

We also observed that older child age was associated with more time spent petting. This observation was of interest in light of the broad consensus in the developmental literature that, beginning in middle childhood, children spend proportionally less of their social time with parents and more time with other social partners (Lam et al., 2012). Research with 7–10 year old children has shown that with age, children broaden their network of social support figures, including pets (Bryant, 1985). With age, intimate disclosure also declines to parents whereas it rises with other social partners such as peers (Buhrmester and Furman, 1987). Although we did not assess peer relationships in this study, the age effected observed is consistent with the notion that non-parental sources of social interaction and support gain in importance during the course of middle childhood, and highlight the role that pets may play in this important developmental transition.

There was no evidence in this study that either genotype or relationship quality was associated with gazing. There are at least two possible explanations for this finding. First, the literature on owner-dog gazing has to date been restricted to research with adults, and there may be unknown differences in childrens’ interactions with their pet dogs compared to adult owners. In the absence of any studies comparing adult to child owner’s interactions with pets, this possibility cannot be ruled out. Second, in contrast to some studies with adults (e.g., Nagasawa et al., 2015), we did not attempt to manipulate owner-dog gazing, but rather quantified the degree to which such behavior naturally occurred in the context of the child soliciting interaction with the dog. It may be that the amount of naturally occurring gazing (approximately 20% of the total interaction time) was too low in our behavioral paradigm to detect association with children’s individual differences.

The present results should be considered in light of several considerations. First, participants were primarily from non-Hispanic White families and thus the generalizability to a more diverse population warrants further study. Second, the participants in this study were typically developing children pre-screened for known health conditions. Whether these findings generalize to clinical populations is unknown; however, the present results may serve as foundational research for application to clinical populations. Third, we did not genotype the pet dogs for variation at the *OXTR* gene. There is some evidence to suggest that dogs’ human-directed behavior is associated with genetic polymorphisms at *OXTR* (Kubinyi et al., 2017; Oláh et al., 2017) or *OXTR* methylation (Cimarelli et al., 2017). Genotyping *OXTR* in both children and the pet dogs may reveal more nuanced associations of *OXTR* genotype within the context of HAIs. Fourth, this study focused on families who already owned a pet dog. This was intentional to avoid the inherent challenge of interpreting child–dog interaction among a mixed group of dog owners vs. non-owners. Finally, the study was conducted in a research laboratory. It is possible that child–dog interaction in a laboratory environment may not be the same as in more naturalistic environments. This limitation is offset, however, by the benefits of the tightly controlled context of a laboratory, with standardized environmental and testing conditions for maximizing the validity of the observed results and reducing distractions and confounding variables. Moreover, the direct behavioral observation of child–pet interaction lends higher confidence in the validity of the observed empirical associations compared to descriptive or self-report studies.

This study adds to the growing body of literature on HAI by documenting two key factors that predict natural variation in children’s interactions with their pet dogs. This knowledge is critical as the field as a whole strives to maximize the potential therapeutic benefits of HAI for clinical populations. A greater understanding of the individual differences that influence children’s interactions with familiar animals will also aid in the broader research goal of determining the potential benefits and challenges of pet ownership for children.

## Author Contributions

DK developed the study, oversaw its execution, analyzed the data, and wrote the manuscript. NH collected the data, conducted the behavioral coding, and analyzed the data. SB conducted the genotyping and analyzed the data. All authors contributed to the writing of the manuscript and approved the final manuscript.

## Conflict of Interest Statement

The authors declare that the research was conducted in the absence of any commercial or financial relationships that could be construed as a potential conflict of interest.
